# Engaging With Communities — Lessons (Re)Learned From COVID-19

**DOI:** 10.5888/pcd17.200250

**Published:** 2020-07-16

**Authors:** Lloyd Michener, Sergio Aguilar-Gaxiola, Philip M. Alberti, Manuel J. Castaneda, Brian C. Castrucci, Lisa Macon Harrison, Lauren S. Hughes, Al Richmond, Nina Wallerstein

**Affiliations:** 1 Family Medicine and Community Health, Duke University School of Medicine, Durham, North Carolina; 2 University of California, Davis, School of Medicine — Internal Medicine, Sacramento, California; 3 Health Equity Research and Policy, Association of American Medical Colleges, Washington, District of Columbia; 4 Director of Community Health, New Brunswick Tomorrow, New Brunswick, New Jersey; 5 de Beaumont Foundation, Bethesda, Maryland; 6 Granville-Vance Public Health, Oxford, North Carolina; 7 Farley Health Policy Center, Aurora, Colorado; 8 Community-Campus Partnerships for Health, Raleigh, North Carolina; 9 Center for Participatory Research, College of Population Health, University of New Mexico, Albuquerque, New Mexico

## Abstract

Coronavirus disease 2019 (COVID-19) has underscored longstanding societal differences in the drivers of health and demonstrated the value of applying a health equity lens to engage at-risk communities, communicate with them effectively, share data, and partner with them for program implementation, dissemination, and evaluation. Examples of engagement — across diverse communities and with community organizations; tribes; state and local health departments; hospitals; and universities — highlight the opportunity to apply lessons from COVID-19 for sustained changes in how public health and its partners work collectively to prevent disease and promote health, especially with our most vulnerable communities.

**Summary**

**What is already known on this topic?**

Responding to pandemics requires engagement with marginalized communities.

**What is added by this report?**

Responding to coronavirus disease 2019 (COVID-19) has demonstrated that effective responses involve partnerships that use a health equity lens, build on community strengths, and use data and community engagement to respond, build trust, and advocate for health for all. Specific steps for effective partnerships are outlined, based on previous recommendations and refined by current examples.

**What are the implications for public health practice?**

Community partnerships are critical elements of public health, and can be built through intentional, stepwise engagement with marginalized communities and wider partners.

## Introduction

Long before the coronavirus disease 2019 (COVID-19) pandemic began, there was widespread recognition of persistent disparities in health outcomes in the United States by race, ethnicity, gender identity, and sexual orientation, as well as awareness that such disparities are symptoms of deeper inequities and racial discrimination across multiple systems and structures. COVID-19 exacerbated these disparities, with Black, Latino, American Indian, and Pacific Islander individuals and their communities having age-adjusted mortality rates 2 or 3 times greater than that of White residents (1). Concerningly, COVID-19’s impact on the lesbian, gay, bisexual, transgender, and queer (LGBTQ) community is largely unknown (2).

Although analysis continues to be hampered by inconsistent collection and reporting of data on race, ethnicity, gender identity, and sexual orientation, possible explanations of COVID-19 disparities include the impracticality or even impossibility of following advice such as physical distancing and self-isolation among those who live in crowded conditions, work in service jobs, cannot telework, or have no sick leave (3). Additional factors affecting some racial/ethnic groups include limited testing availability and mistrust of accessing testing in some racial/ethnic communities once testing is offered (4); the need for communications in languages other than English; failure to provide protective equipment to essential workers, who are often from specific racial/ethnic groups; and closures of work places that disproportionally impact some racial/ethnic communities, leaving increasingly large numbers without employer-sponsored health insurance (1,5,6). LGBTQ Americans report difficulty accessing needed treatments, and most are concerned about the combined risks of COVID-19 and HIV (7). These factors are compounded among the homeless or those who are incarcerated (8). Social stigma and racism are factors as well. Black men are reportedly less likely than White men to wear face coverings out of fear of police harassment and violence (9). In addition, Black men who violate stay-at-home orders in 3 of the most populous jurisdictions in Ohio (Toledo, Columbus, and Cincinnati) are 4 times more likely than White men to be charged for violating the orders (10). Finally, a long-term mistrust of government, research, and health care institutions, built on decades to centuries of neglect and abuse, including but far from restricted to the Tuskegee syphilis study (11), make it less likely that some racial/ethnic communities and historically marginalized communities will trust public health messaging by these bodies, or will believe that they will receive equal access to testing, treatment, and vaccines (12).

Despite and often because of these realities, communities, local health departments, and partners across the country with histories of collaboration were able to rapidly react to the challenges of COVID-19. By using community-engaged/participatory research and programmatic coalitions to showcase and bolster the resiliencies within communities and across partnerships, they were able to respond to immediate and critical needs. Here are a few early examples:

- In Chicago, the Homelessness and Health Response Group for Equity coalesced multiple working groups into a coalition of more than 100 members, including hospitals, federally qualified health centers (FQHCs), city officials, shelter operators, housing advocates, and others. Meeting every morning, they established dedicated quarantine and isolation sites for people with unsafe home environments in which to self-isolate; acquired and distributed tens of thousands of pieces of donated personal protective equipment to group settings across the city; crafted evidence-based guidance for varied settings; administered tests to thousands of individuals; created housing for those who were healthy yet at high risk so as to shield them from ongoing outbreaks; and established clinical linkages for shelters and with FQHCs to provide outreach and health checks for high-risk groups (13).
- The University of California, San Francisco (UCSF), leveraged expanded test processing at UCSF and partnered with community organizations to test all residents of a densely populated portion of San Francisco’s Mission District as well as the small, rural town of Bolinas. This community-wide testing effort began as a grassroots initiative in Bolinas, driven by residents who partnered with UCSF scientists, state and county public health departments, and the local Coastal Health Alliance, to ensure community engagement and support (14).
- The Navajo Nation, among other tribes, is facing some of the worst rates of COVID-19 in the United States. The tribe has long-standing health inequities attributable to persistent federal neglect, a high prevalence of chronic disease, and geographically dispersed multigenerational homesteads, often with no running water or internet access. As COVID-19 struck, Navajo Nation President Jonathan Nez immediately created a Health Command Center, working with state and local governments, the Indian Health Service, and hospitals to begin testing and contact tracing. With the delay of release of federal funds to tribes, Navajo launched its own COVID-19 Relief Fund (15), and local nonprofits and GoFundMe efforts stepped up, distributing food and medical and household supplies, with volunteers dropping off boxes to families with someone positive for COVID-19 and in self-quarantine. The Gallup-based Community Outreach and Patient Empowerment organization (16), a partnership with the Navajo Nation, Brigham and Women’s Hospital, and Partners in Health, and the nonprofit United Natives (17) coordinated medical supplies for clinicians and home-based resources for community members; the Na’nizhoozhi Treatment Center and the City of Gallup provided needed housing; and Auntie Project, Native women from Oklahoma, sent peer-to-peer financial support. Long-term academic partners contributed: for example, the Johns Hopkins Center for American Indian Health organized 140 Native American and other health professionals for surveillance, education, and critical supplies; the University of California, San Francisco, and Doctors Without Borders sent volunteer clinicians; and the University of New Mexico’s Transdisciplinary Research, Equity and Engagement Center for Advancing Behavioral Health (TREE Center) health equity center worked with the Diné Centered Research and Evaluation Group and provided material and emotional support (18).
- A partnership across the University of New Mexico, city of Albuquerque, state and city emergency operations centers, nonprofits, primary health care clinics, the city department of health, and the Medical Reserve Corps used the community-based participatory research (CBPR) model as a planning and evaluation tool (19). The partnership first identified a short-term goal of encouraging homeless people, especially older adults, not to leave shelters. To strengthen engagement of seniors unused to sheltering in place, the partnership created a rapid-cycle CBPR process of surveying seniors on their perceived barriers to staying at the shelter, returning the results through town hall dialogues, then providing COVID-19 testing within 2 days, and responding to recommendations, such as increased meal variety, more activities, toiletries and snacks, and improved access to medical providers and case managers. After the first 3 weeks, the proportion of seniors who stayed in the shelter after sleeping there grew from 20% to 75%, with no one testing positive for COVID-19. As the crisis continued, new goals were established each week, with responses including hotel rooms paid for by the city and state for people with COVID-19, and contact tracing for this difficult-to-reach population (20).
- LGBTQ communities have organized information networks and support funds as well as advocated for the needs of LGBTQ communities (21). In semirural Solano County, California, the Solano Pride Center is conducting virtual emotional support and practical information sessions for LGBTQ youth and older adults and has opened a chat service and other safe spaces in response to the social isolation and limited emotional support accentuated by the COVID-19 crisis (22).
- In rural Eagle County, Colorado, the response built on the Mobile Intercultural Resource Alliance, which serves as a clearinghouse for local services in health education and screenings, application support for public assistance programs, food resources, workforce development, early childhood education coordination, and physical activity programming. Funded by Vail Health, Eagle Valley Community Foundation, and Eagle County government, and housed in a recreational vehicle that travels from community to community, it brings needed services to low-income and often isolated communities in the region. As schools closed, they shifted to providing information, free COVID-19 tests, and school lunches to anyone who needs one (23).
- In New Brunswick, New Jersey, the response has been channeled through peer-to-peer interaction and networks of partnerships with a history of practicing collective impact. Community health ambassadors, New Brunswick residents who decided to do their part to better their community, serve as the cultural bridge between community-based organizations, health care agencies, and their respective communities. They have provided valuable community insight during the pandemic. They, along with the New Brunswick Heathy Housing Collaborative partners (New Brunswick Tomorrow, Robert Wood Johnson University and Saint Peter’s University hospitals, and the Middlesex County Office of Health Services) are part of a multisector network (Healthier New Brunswick) that has continued to work together to mitigate the effects of COVID-19 (24). Saint Peter’s University Hospital conducted an informal geo-mapping of infected New Brunswick residents and found that close to 100% of New Brunswick residents infected with COVID-19 lived in 2 predominately Latino neighborhoods whose census tracts have the most substantial health and social disparities in the city. In response, the hospitals put together care kits that included masks, soap, and public service announcements (in English and Spanish) on proper prevention methods, which the hospitals and community partners disseminated in these neighborhoods. Other announcements addressing COVID-19 health concerns and underlying structural inequities (inability to isolate in home settings) are promoted by using community outreach and New Brunswick Tomorrow’s health communications initiative (Live Well Vivir Bien New Brunswick) that uses a website, mobile app, and social media outlets.
- The state of North Carolina, recognizing the impact of COVID-19 on its racial/ethnic communities and the substantial challenge of contact tracing efforts by its local public health departments, partnered with its state primary care Medicaid program, Community Care of North Carolina (CCNC), and the North Carolina Area Health Education Centers to hire and train staff to augment local health department–led efforts in tracking transmission (25). CCNC has worked with and within local health departments for more than decade, supporting and improving data standardization for the statewide care management services provided for children and pregnant women. The need for food and housing security has been amplified in poor, rural areas of the state during the isolation and quarantine efforts of the pandemic response, so the state also accelerated the rollout of NCCARE360, an electronic coordinated care network to connect those with identified needs to community resources and allow for a feedback loop via electronic health record or web-based notifications on the outcome of those connections (26). The personal care management provided to individuals locally, in concert with the new technologically advanced data system, aims to facilitate the connection of individuals to badly needed services and resources.
- In the coastal plain town of Raeford, North Carolina, Dr Karen Smith, a solo family practitioner, was called by her local health department director about a potential outbreak at a 24-bed youth treatment center, where 2 staff members had tested positive. A quick call to First Health, the local hospital, yielded testing kits; testing was quickly accomplished, and the local emergency medical services drove the tests to Raleigh. Fourteen were positive, and the facility then was able to separate, isolate, trace, treat, and monitor both positive and negative cases (27).

Academic groups have stepped up as well:

- Historically Black colleges and universities (HBCUs) are at the epicenter of large-scale outbreaks. Howard University partnered with Wells Fargo to offer free testing in Ward 7 of Washington, District of Columbia (which had among the highest rates in the Maryland, District of Columbia, and Virginia region) (28). North Carolina Central University (NCCU), with Duke University and University of North Carolina at Chapel Hill, are partnering with Granville Vance Public Health to offer free mobile testing in rural communities in northeastern North Carolina, with a special focus on Black and Latino neighborhoods and churches. And NCCU, along with 5 other HBCUs, was just awarded state funds to study the public health and economic impact of COVID-19 in the state’s underserved communities (29).
- Schools of public health have taken lead roles in analysis and advice responses locally and nationally (30).
- Multiple medical schools and health centers have responded, especially those with histories of community engagement. The Center for Reducing Health Disparities at the University of California, Davis, quickly became a local resource and coordination point for community-engaged efforts, especially in Latino communities and for those with behavioral health challenges (31). The HealthStreet Community Engagement Program at University of Florida, which has been working to build community trust, pivoted from being a face-to-face community health worker model to a telephone-based program to continue to assess the needs of their 12,000 members and link them to needed services (32). In Minnesota, a community-engaged research partnership worked with community leaders to refine messages, leverage resources, and advise policy makers on a community-based risk communication framework, which was used to deliver messages in 6 languages across 9 electronic platforms to almost 10,000 individuals over 14 days (33).
- Nursing schools have engaged, including offering resources for health equity (34).
- Hundreds of public health, medical, nursing, and other students have participated in local public health activities, including serving as contact tracers (35).

## Lessons From the Past

Partnering with the community and collaborating with its members have long been recognized as cornerstones of efforts to improve public health and its core value of social justice. Community engagement was a critical driver of success during the AIDS epidemic, when activists raised awareness, educated individuals about strategies to reduce their risk, and advocated for timely governmental response. Community-based organizations in racial, ethnic, and sexual communities played critical roles in HIV prevention efforts, as the Centers for Disease Control and Prevention (CDC) recognized that such efforts “must be appropriate for and responsive to the lifestyle, language, and environment of members of that population” (p. 704) (36).

These lessons were reinforced in 1995, when CDC, recognizing the importance of involving the community, established the Committee for Community Engagement, which was composed of representatives from across CDC and the Agency for Toxic Substances and Disease Registry (ATSDR). That committee developed the booklet Principles of Community Engagement, which was published by CDC and ATSDR. A second, enlarged edition of the Principles of Community Engagement was published in 2011 by the National Institutes of Health (NIH), with CDC and ATSDR (37). The same year, CDC released its social vulnerability index, facilitating the ability of local officials to identify communities that may need support in responding to hazards (38).

The response to the next major outbreak, severe acute respiratory syndrome (SARS) in 2003, again noted the need to identify high-risk groups; provide close, targeted communication and coordination across community partners; and ensure access to needed supplies by those in isolation or quarantine (39).

The Institute of Medicine (IOM) report The Future of the Public’s Health in the 21st Century reinforced the idea that public health’s broad mission of ensuring healthy communities required interactions among numerous health-influencing actors, such as communities, businesses, the media, governmental public health, and the health care delivery system (40).

These reports were accompanied by a broader movement of agencies in partnering with communities in improving health. In 2006, NIH established the Clinical and Translational Science Awards to spur clinical and translational research, with community engagement as one of its core functions. The IOM reinforced this effort in a 2013 review of the program, calling for ensuring community engagement in all phases of research (41). Similar efforts followed in the National Institute on Minority Health and Health Disparities (42) and National Institute on Drug Abuse (43).

A parallel IOM initiative in 2012 assessed the opportunity to link primary care and public health around the needs of communities, noting that “Improving population health will require activities in 3 domains: 1) efforts to address social and environmental conditions that are the primary determinants of health, 2) health care services directed to individuals, and 3) public health activities operating at the population level to address health behaviors and exposures” (p. 19) (44). In turn, this led to the establishment of a collaboration between the deBeaumont Foundation, CDC, the Health Resources and Services Administration, and Duke University to provide practical tools for partnerships for health, to connect interested individuals and organizations, and to support training and capacity building for partnerships for health (45).

Common across all these examples and activities are several principles, which have been consistent themes for how public health and its partners can effectively engage to ensure improved health in diverse communities (37):

- “All aspects of community engagement must recognize and respect the diversity of the community. Awareness of the various cultures of a community and other factors affecting diversity must be paramount in planning, designing, and implementing approaches to engaging a community” (p. 51).
- “Partnering with the community is necessary to create change and improve health” (p. 50).
- “Organizations that wish to engage a community as well as individuals seeking to effect change must be prepared to release control of actions or interventions to the community and be flexible enough to meet its changing needs” (p. 52).

## Public Health Implications

Pandemics and epidemics are most dangerous to those already at risk: people with underlying health conditions (caused, in part, by deeper racial, structural, and systemic inequities), and those who are members of marginalized communities without access to preventive care or health care services at their time of greatest need. As was seen in AIDS, SARS, and now COVID-19, responding to an evolving pandemic requires identification of and collaboration with those groups at greatest risk, who often lie outside the mainstream. Engagement with communities early on and throughout is critical, especially communities of color and other marginalized groups that require a public health response that is not channeled through discriminatory systems and structures and does not perpetuate inequities in the midst of crisis. Effective public health roles include gathering data on those affected; building from community strengths and priorities to shape the actions of collecting, sharing, and interpreting data with the communities; developing plans with community leaders; co-creating and communicating risk and harm reduction strategies through existing communication methods; and rapidly tracking and adjusting plans as the epidemic progresses. Although public health holds a leadership role during the epidemic response, it needs the engagement, partnerships, and trust of communities in shaping, communicating, implementing, and disseminating recommended strategies. Trust can only be built when government and academic collaborators are themselves trustworthy and engage communities as partners in addressing what matters to them, including inequities in testing, treatment, and potentially future access to vaccines. Community engagement and partnerships are at the heart and core of public health, are essential for achieving health equity, and are most dramatically needed during pandemics such as we now face.

The Box outlines practical steps that public health can take to successfully engage with its communities and partners for sustained equitable changes in how we live, learn, work, and play. What is not known, but which COVID-19 is helping us learn, is what additional steps public health and its partners can take to effectively work together so that trust is established and maintained, resilience is strengthened, and communication plans are refined. We must also learn how to effectively communicate the need for long-term investment in the infrastructure required for healthy, productive communities, including public health, health care from primary care through hospitals, and community partners. COVID-19 is not our last disaster, and the lessons (re)learned can both prepare us for the next challenge and help reduce and eliminate our long-standing underlying inequities in health.

**Box. Steps That Public Health Can Take to Engage With Communities and Partners for Sustained Changes in How We Live, Learn, Work, and Play**

**Learn**

- Train staff in health equity, using local resources or national training such as the National Association of County and City Health Officials’ online course Roots of Health Inequity (46)
- Learn about effective multisector partnerships through sources such as The Practical Playbook (45)
- Reframe the COVID-19 pandemic as a “community” problem in which social determinants of health play leading roles, not just a “public health” problem

**Partner**

- Gather, share, and interpret data with affected communities, working with community members and leaders, and with analysis by race, ethnicity, language, location (zip code or census tract), and social factors
- Identify the unique risks and protective factors with affected communities
- Ensure equitable access to testing, protective equipment, clinical trials, and treatment
- Incorporate community oversight as a quality assurance tool

**Work collectively (47)**

- Design and implement with a priority placed on equity
- Co-create with cross-sector partners — community-based organizations, clinicians, universities, medical centers, schools of public health (especially those located in or partnered with racial/ethnic communities), housing and transportation sectors, and community development, among others. Students, including in public health, medicine, and nursing, have much to contribute and learn
- Collectively define the problem and create a shared vision to solve it
- Focus on outcomes — not just on activities or processes
- Use data to continuously learn, adapt, and improve
- Develop and deliver health risk messaging that is culturally and linguistically appropriate, relevant to vulnerable communities, and delivered through trusted sources (48)
- Move beyond information delivery to community conversations that encompass knowledge, beliefs, attitudes, and behavior
- Build a culture that intentionally fosters relationships, trust, and respect across participants

**Share**

- Gather and distribute stories and data both of initial failures and of solutions found
- Participate in a learning collaborative, such as Community Campus Partnerships in Health’s Communities in Partnership: Ensuring Equity in the Time of COVID-19 (49), and the Big Cities Health Coalition (50)

**Advocate**

- Engage with partners in coordinated efforts to advocate for immediate support for communities that are most affected, for removal of barriers, for support of programs that address the root causes of health inequity, and for a diverse public health and health care workforce that works together in partnership with its communities
- Pursue health in all policies as a fundamental tool for ensuring health for all (51)
